# Supplementary material for: The p300/YY1/miR-500a-5p/HDAC2 signalling axis regulates cell proliferation in human colorectal cancer
Source: Nat Commun. 2019 Feb 8;10:663. doi: 10.1038/s41467-018-08225-3 (PMC6368584; doi:10.1038/s41467-018-08225-3)
Supplement: Supplementary file 1 — Supplementary Information [file 41467_2018_8225_MOESM1_ESM.pdf]

Supplementary Information

Title：The p300/YY1/miR-500a-5p/HDAC2 signalling axis regulates cell proliferation in human colorectal cancer

Tang，W et al

Supplementary Fig 1

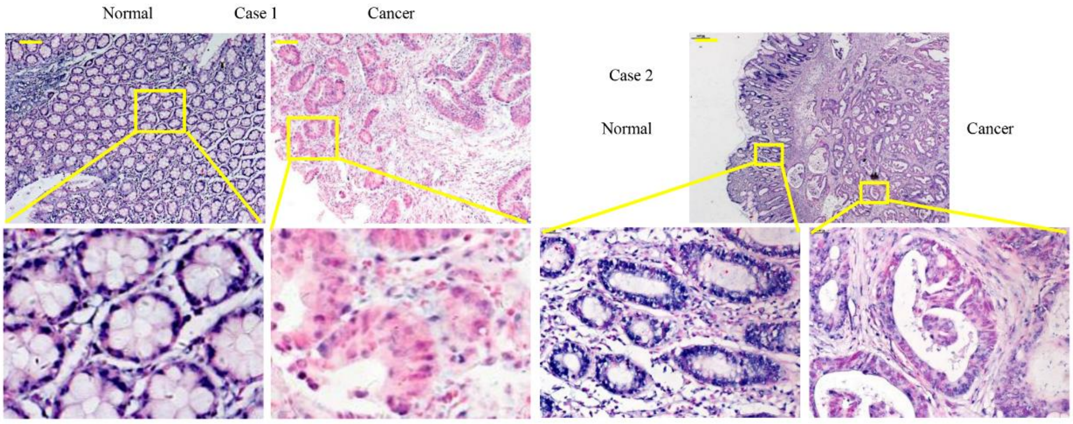

Supplementary Fig. 1 MiR-500a-5p expression was detected in CRC and adjacent normal tissue by in situ hybridization (ISH). Scale bars, 50 μm.

Supplementary Fig 2

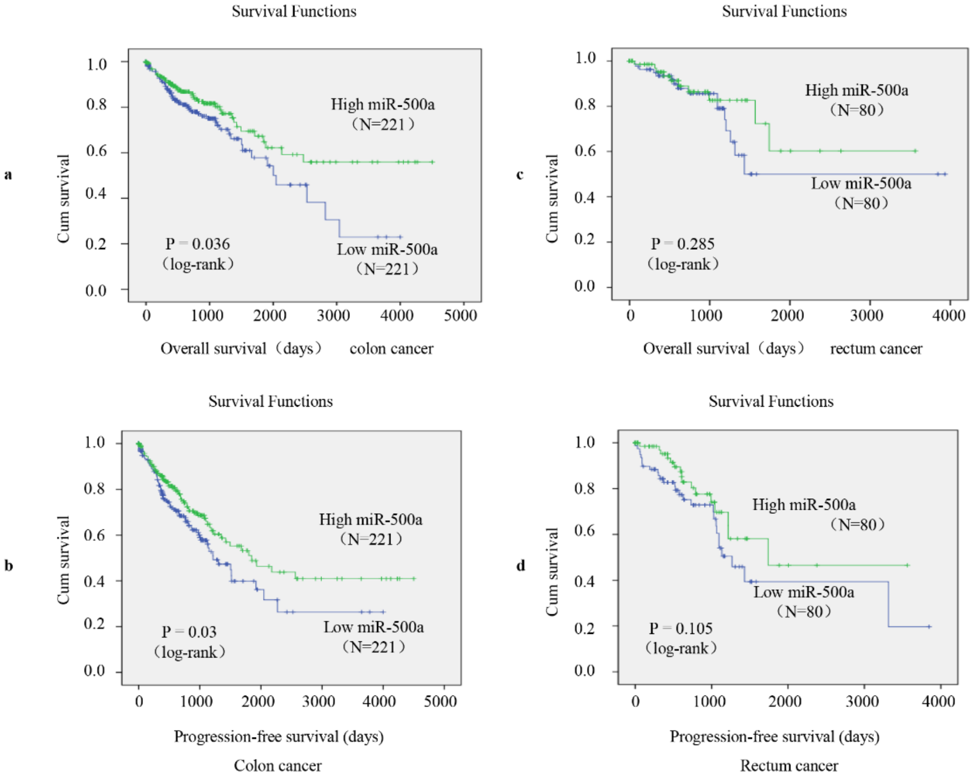

Supplementary Fig. 2 Kaplan-Meier curves for overall survival (OS) (A and C) and progression-free survival (PFS) by (b and d) miR-500a-5p expression. Log-rank test.

Supplementary Fig 3

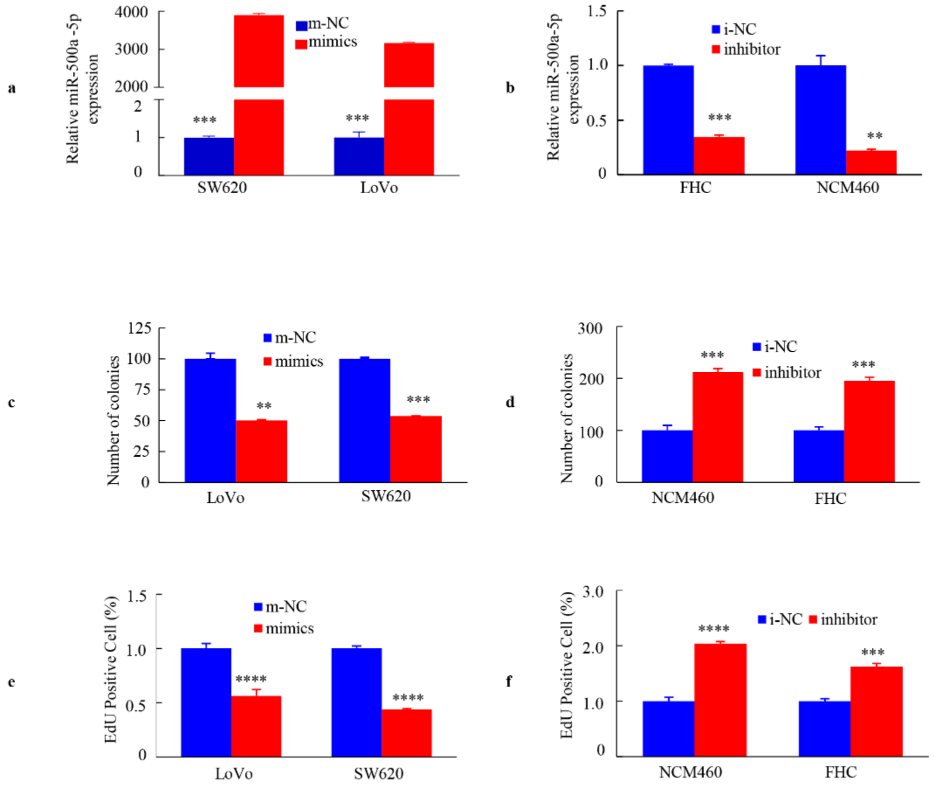

Supplementary Fig. 3 The effect of miR-500a-5p in CRC cells on cell proliferation in vitro. (a) & (b) MiR-500a-5p expression following transfection of colon epithelial cells, as confirmed by qRT-PCR. Student's t-test. ** P < 0.05; *** P < 0.01. **(c) & (d)** Effects of miR-500a-5p mimics or inhibitor on the proliferation of colon epithelial cells, as determined by colony formation assay. Student's t-test.**P < 0.05 and *** P < 0.01. **(e) & (f)** DNA synthesis in colon epithelial cells was measured by the EdU incorporation assay. Student's t-test.****P < 0.001, mimics vs. m-NC; and *** P < 0.01 and ****P < 0.001, inhibitor vs. i-NC. All results are expressed as the mean of three independent experiments ±SD.

Supplementary Fig 4

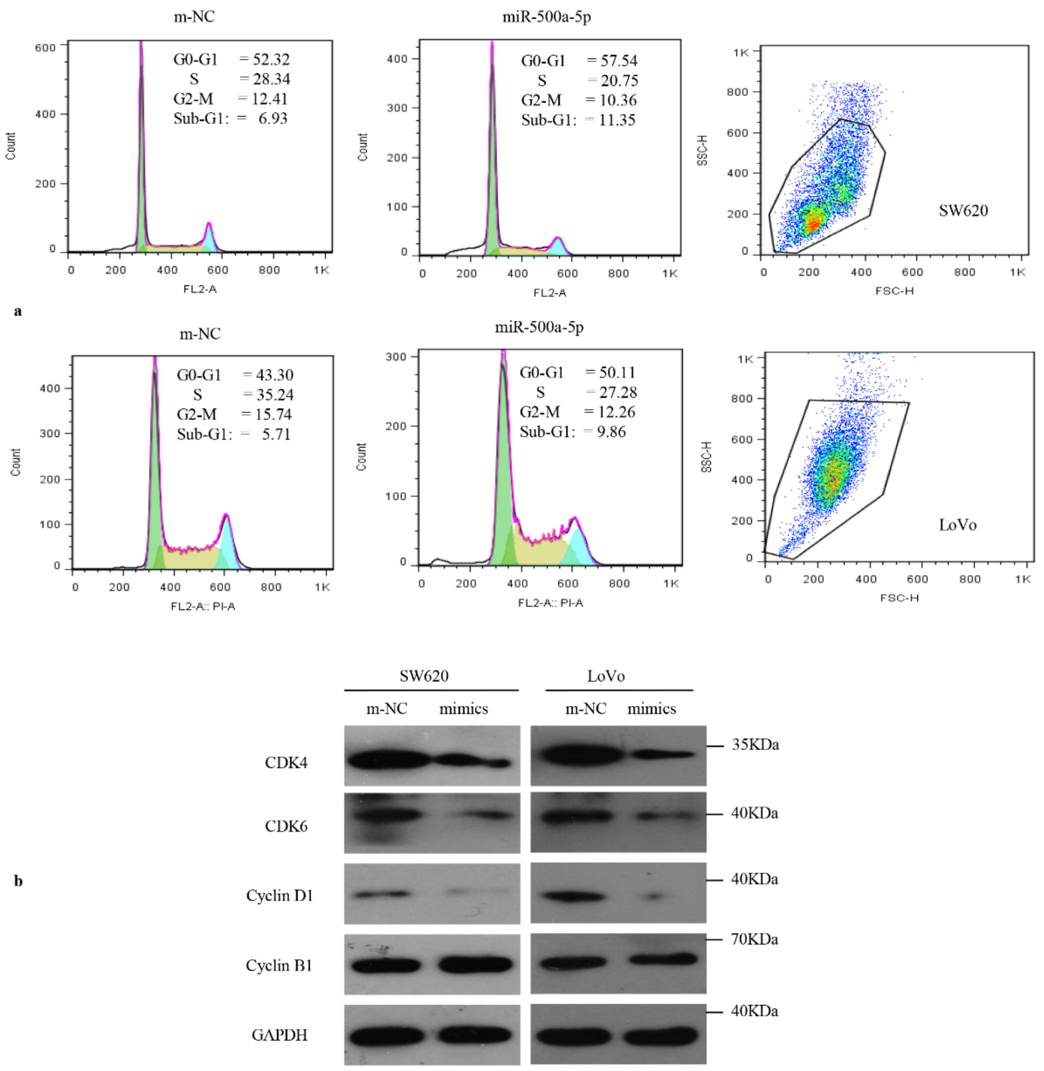

Supplementary Fig. 4 The effect of miR-500a-5p on cell cycle progression of CRC cells. **(a)** The effects of miR-500a-5p (mimics) or m-NC on the cell cycle distribution of CRC cells were determined by flow cytometry after staining with PI. The R1 gate is determined by forward-scatter area (FSC) and side-scatter area (SSC). **(b)** The expression of cell cycle related proteins was detected in CRC cells. These figures are representative of three independent experiments.

Supplementary Fig 5

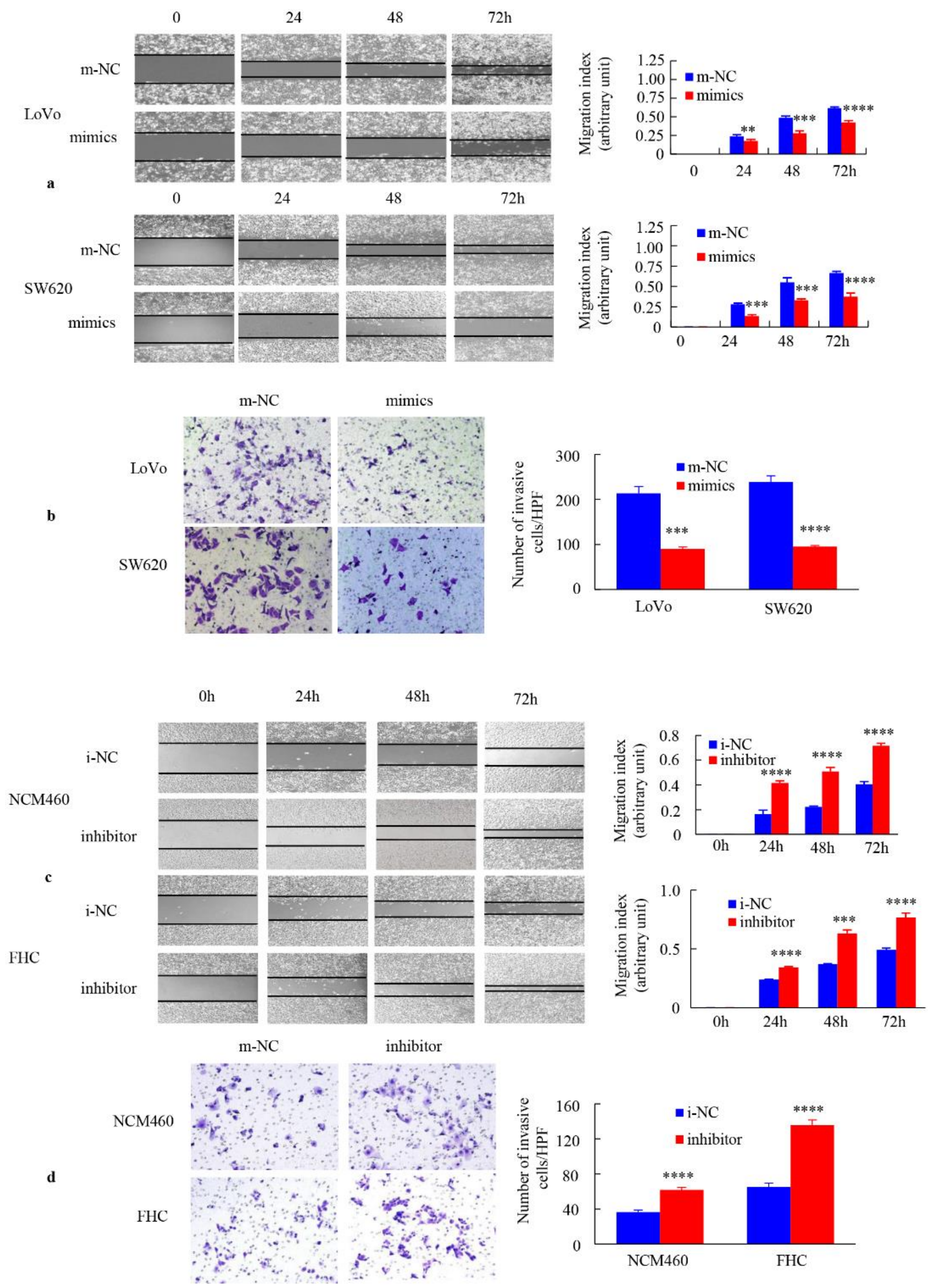

Supplementary Fig. 5 The effect of miR-500a-5p on the metastatic and invasive capacity of colon epithelial cells. (a) & (c). Cell migration activity was measured by the wound healing assay. Student's t-test .**P < 0.05; *** P < 0.01;**** P < 0.001. **(b) & (d).** Cell invasive activity was measured by the transwell assay. Student's t-test . *** P < 0.01; **** P < 0.001. The experiments were repeated three times with similar results.

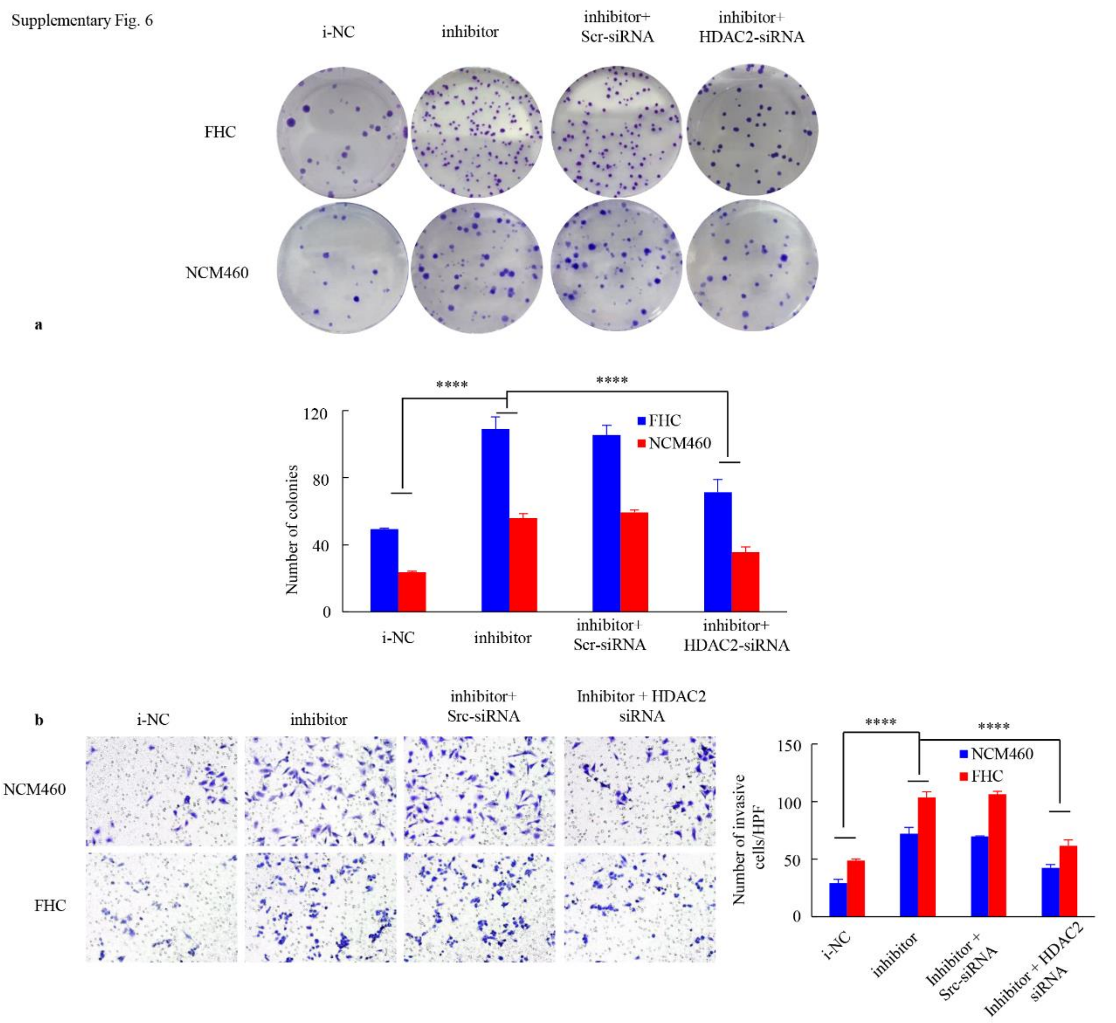

Supplementary Fig. 6 HDAC2 is a direct downstream target of miR-500a-5p. (a) Representative results (up) and quantification (down) of crystal violet-stained cell colonies formed by the indicated colon epithelial cells lines at 12 days after seeding. Student' s t-test .**** P < 0.001, i-NC vs. inhibitor and inhibitor plus scr (scrambled) siRNA or inhibitor plus HDAC2 siRNA, respectively. **(b)** The invasive activity of cells transfected with i-NC, inhibitor, inhibitor plus src siRNA or inhibitor plus HDAC2 siRNA, respectively. Student's t-test .**** P < 0.001, i-NC vs. inhibitor and inhibitor plus scr siRNA or inhibitor plus HDAC2 siRNA, respectively. The experiments were repeated at least three times.

Supplementary Fig. 7

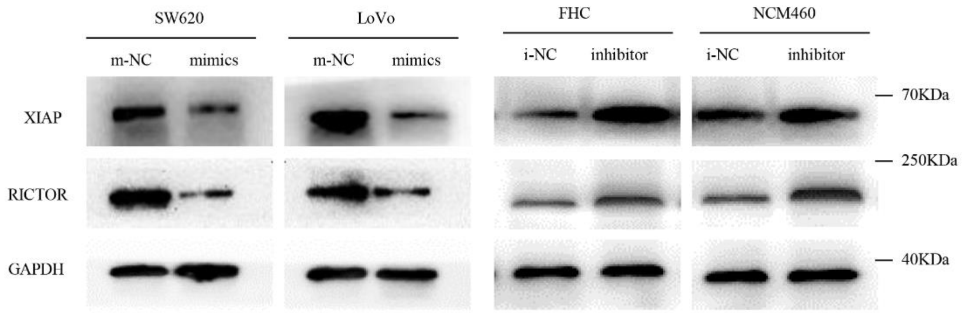

Supplementary Fig. 7 MiR-500a-5p target XIAP or RICTOR in CRC cells. Western blot analysis of XIAP or RICTOR protein expression in SW620 and LoVo cells transfected with miR-500a-5p mimics. Transfected cells with an m-NC plasmid were used as controls. The experiments were repeated three times with the same results.

Supplementary Fig. 8

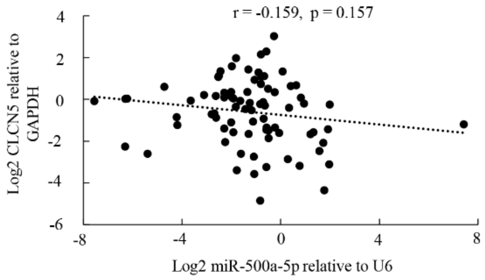

Supplementary Fig. 8 The expression of *miR-500a-5p* and host genes *CLCN5* in CRC tissues. MiR-500a-5p expression uncorrelated with CLCN5 expression in 81 CRC tissues by q-PCR. Linear regression analysis, r=-0.159 * P = 0.157. All of these experiments were repeated three times with identical findings.

Supplementary Fig. 9

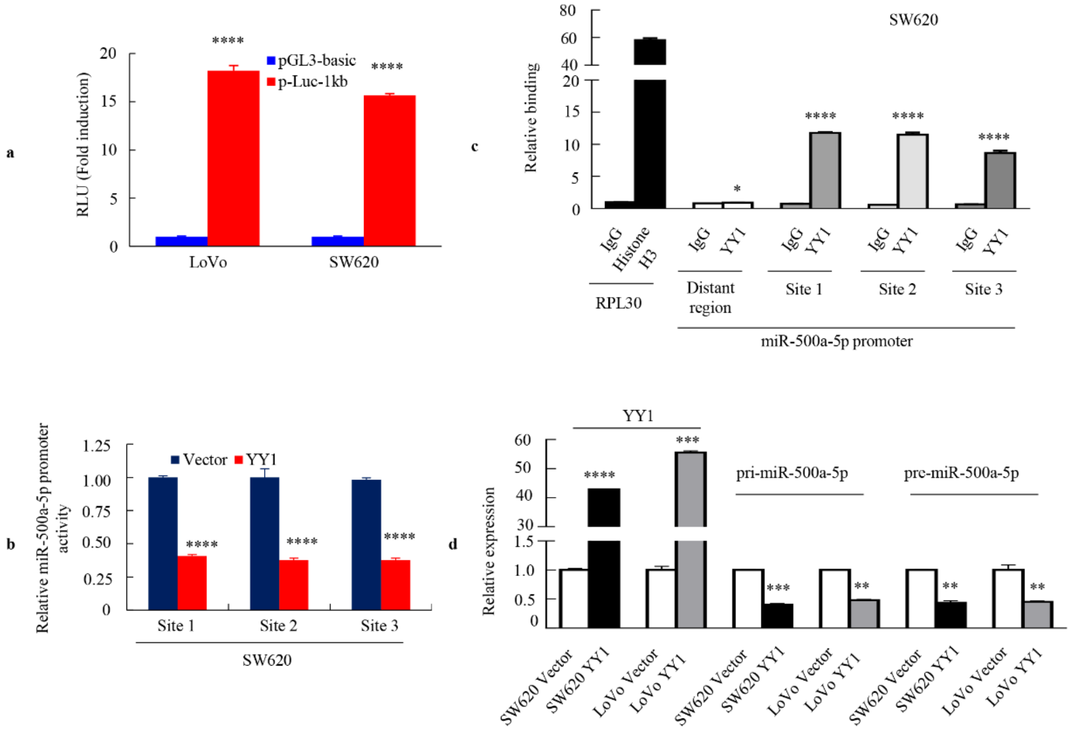

Supplementary Fig. 9 miR-500a-5p is regulated directly by the transcription factor YY1. (a) The CRC cells were transiently transfected with pGL3 basic plasmid, or the p-Luc-1kb vector of the putative promoter region of miR-500a-5p upstream of the luciferase reporter plasmid for 48 h. Promoter activity was presented as the fold induction of RLU as compared with pGL3 basic plasmid. RLU = values of firefly luciferase unit/values of *Renilla* luciferase unit. This result is expressed as the mean of three independent experiments ±S.D. Student's t-test . ****P<0.001. **(b)** Luciferase activity of the miR-500a-5p promoter construct after transfection of YY1 or vector plasmid in SW620 cells. Student's t-test. ****P < 0.001. **(c)** A ChIP-qPCR assay demonstrated the direct binding of YY1 to the miR-500a-5p promoter in SW620 cells. Fold changes are normalized to the IgG. Student's t-test. *P > 0.05; ****P < 0.001. **(d)** Expression of YY1, primary (pri-) and precursors (pre-) miR-500a-5p in YY1-

overexpressing CRC cells measured by q-PCR. Data was normalized to vector. Student's t-test. ** P < 0.05; ***P < 0.01; ****P < 0.001. These figures are representative of three independent experiments.

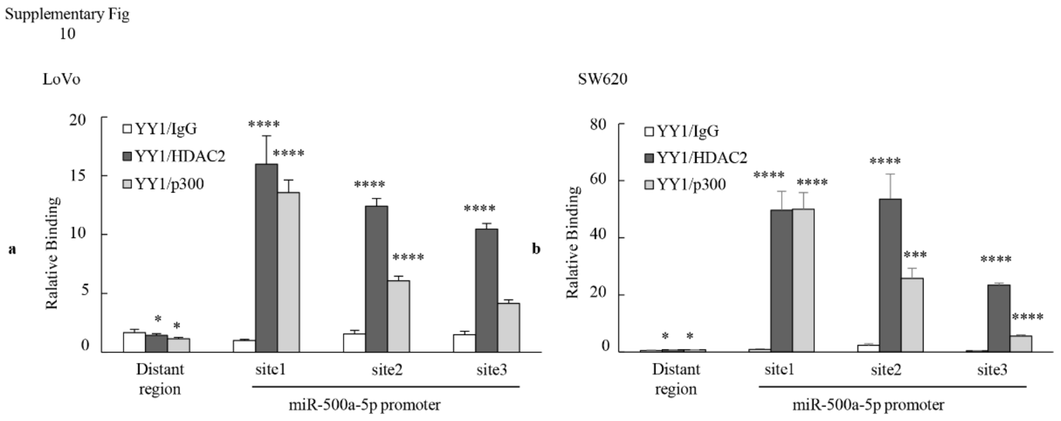

Supplementary Fig. 10 YY1-HDAC2 or YY1-p300 interaction on miR-500a-5p promoter in re-ChIP. Chromatin was precipitated with anti-YY1 antibody, and re-precipitated with anti-HDAC2 or p300 antibody or IgG, followed by q-PCR in LoVo (A) or SW620 (B) cells. Student's t-test.*P > 0.05; ***P < 0.01; ****P < 0.001. All of these experiments were repeated three times with identical findings.

Supplementary Fig 11

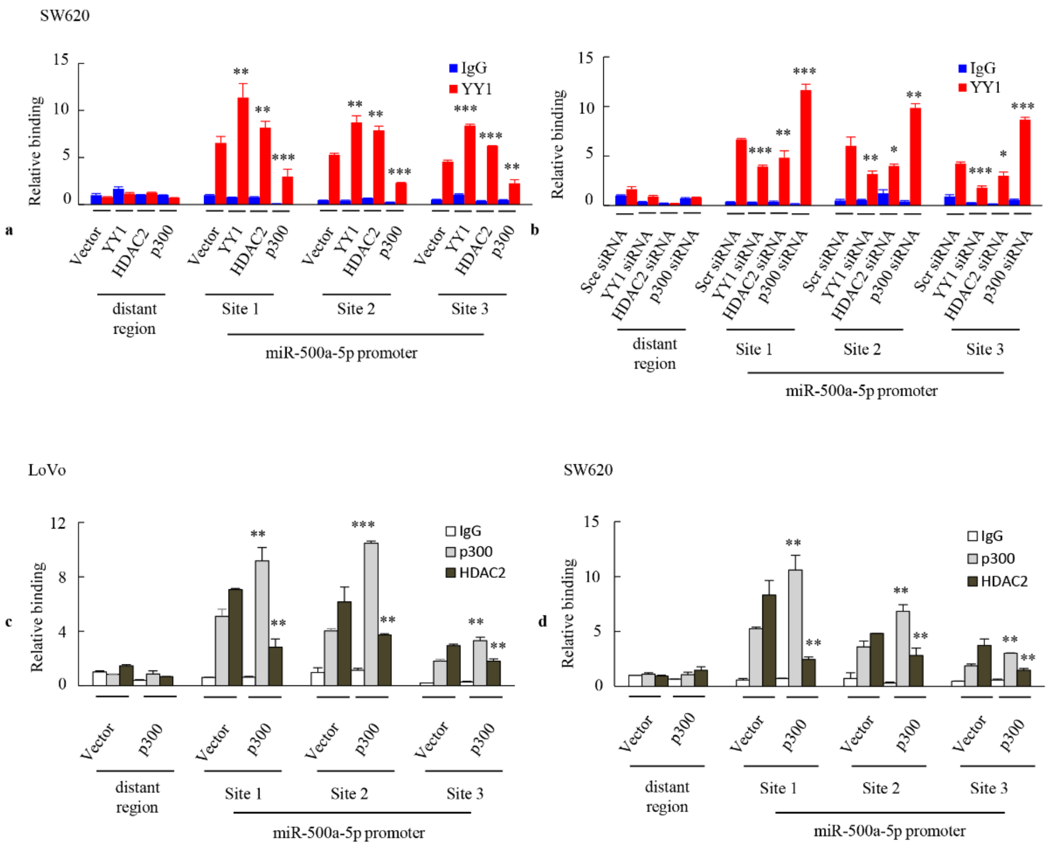

Supplementary Fig. 11 YY1, p300 and HDAC2 form a complex regulate the miR-500a-5p promoter in CRC cells. (a) & (b) Analysis of YY1 binding to various miR-500a-5p promoter regions after overexpression of YY1, HDAC2 or p300. Student's t-test. ** P < 0.05 and *** P < 0.01 between vector and gene. **(c) & (d)** Forced expression of p300 down-regulated HDAC2 binding to the miR-500a-5p promoter region. Student's t-test. ** P < 0.05 and P < 0.01 between vector and p300. Results of experiments were repeated three times with the same results.

Supplementary Fig
12

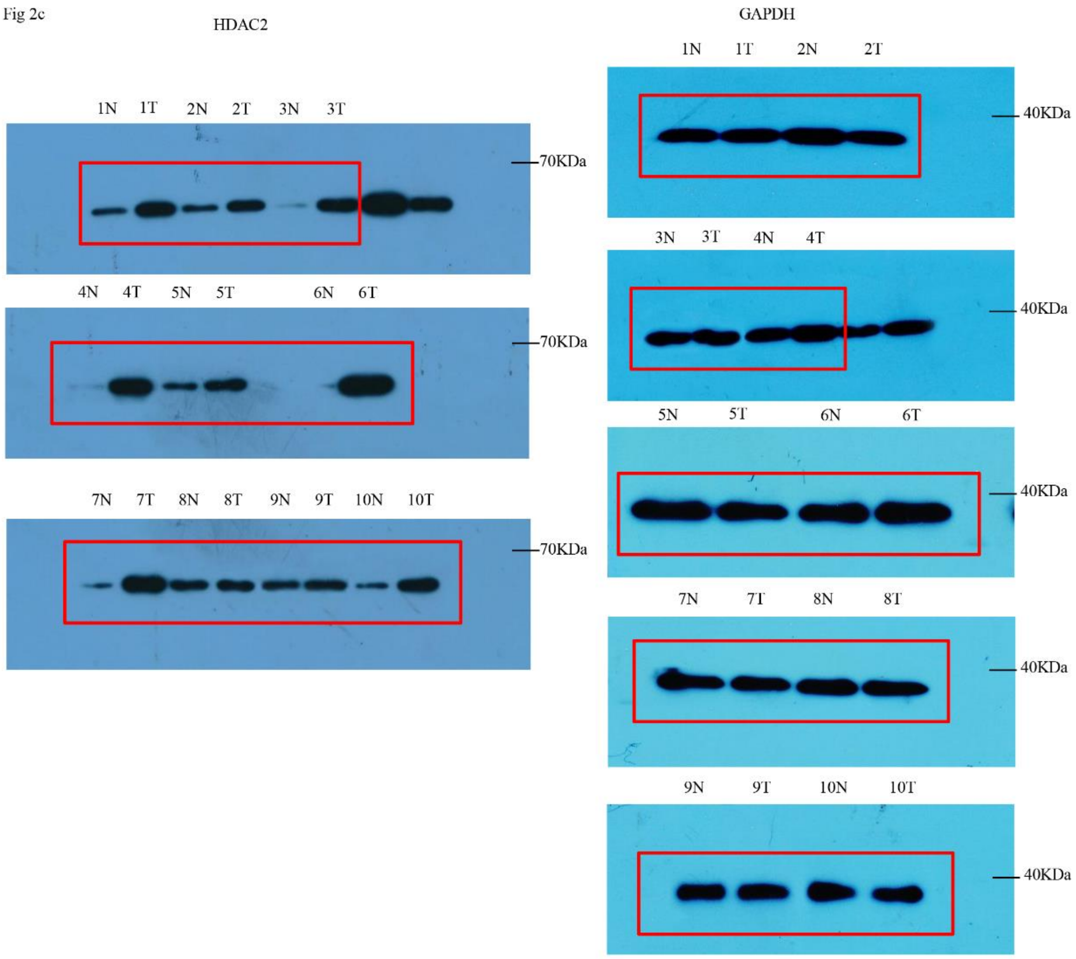

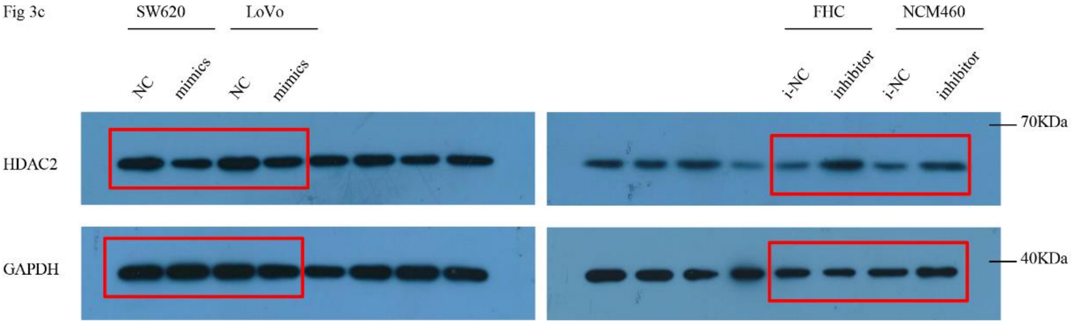

Supplementary Fig. 12 Original Western blotting images of the Fig 2c and Fig 3c.

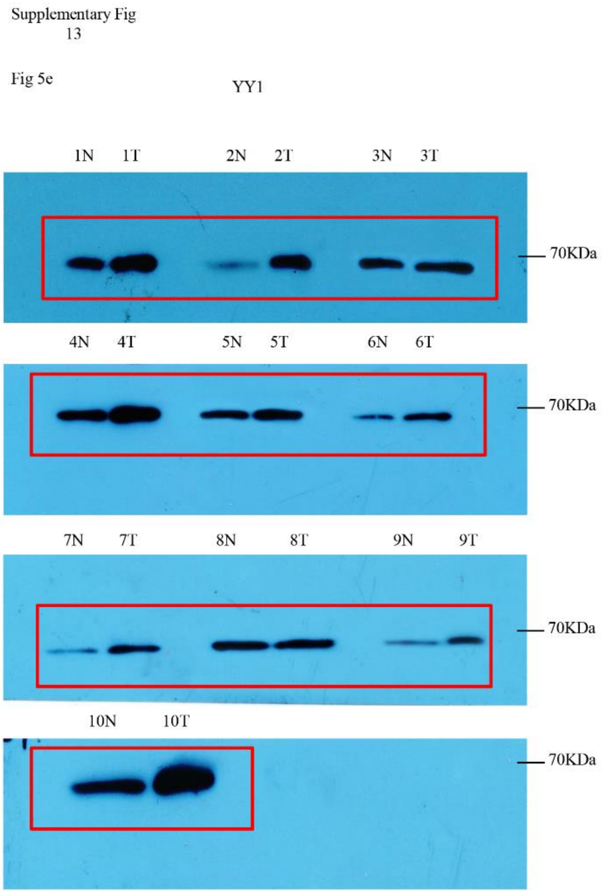

Supplementary Fig. 13 Original Western blotting images of the Fig 5e.

Supplementary Fig
14

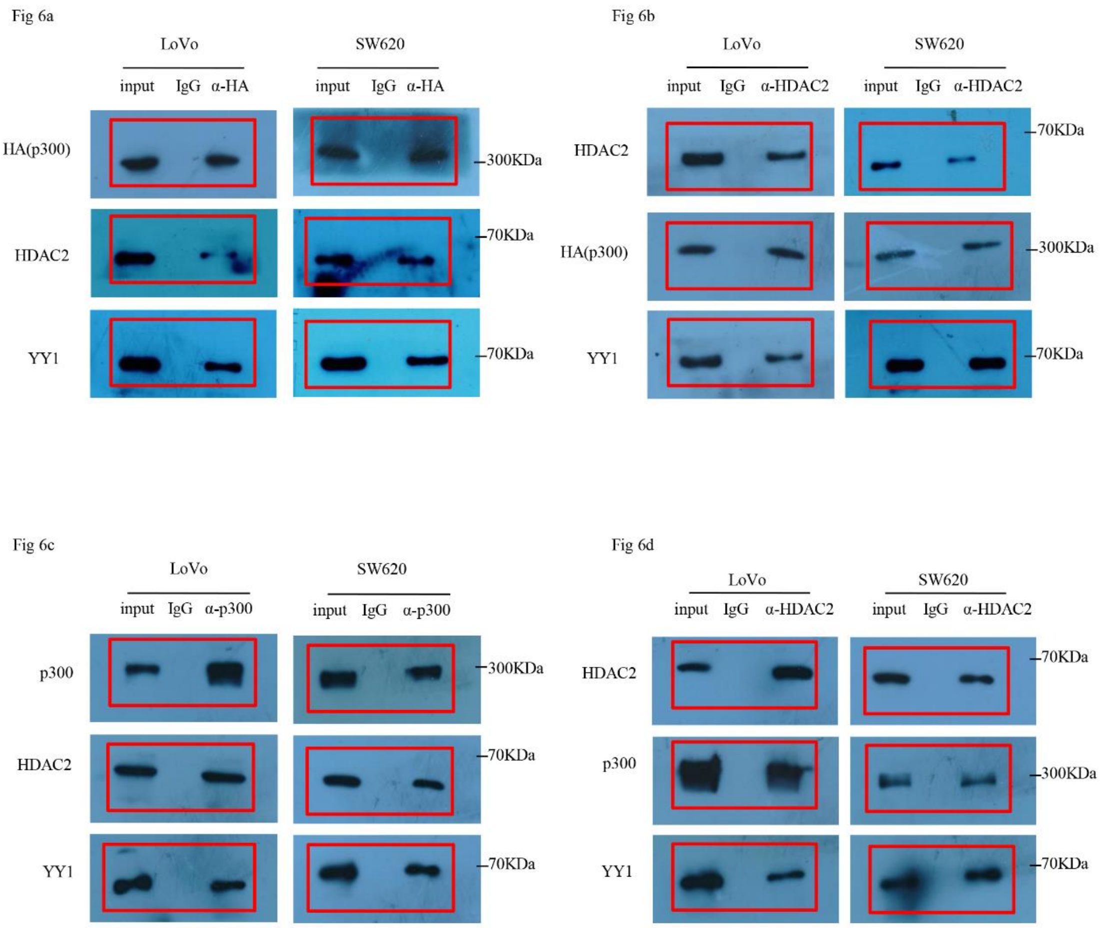

Supplementary Fig. 14 Original Western blotting images of the Fig 6a~d.

Supplementary Fig
15

Supplementary Fig 4b

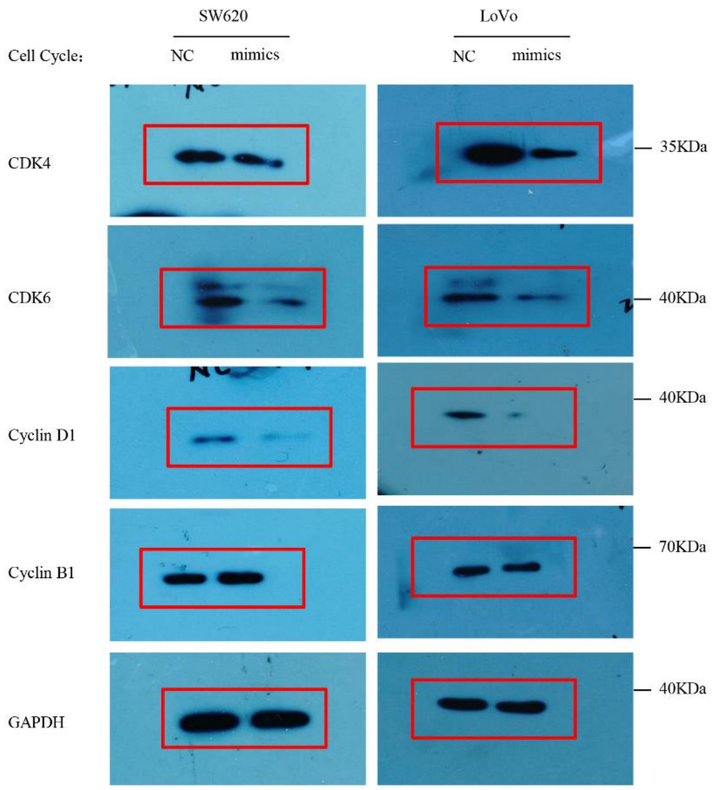

Supplementary Fig 7

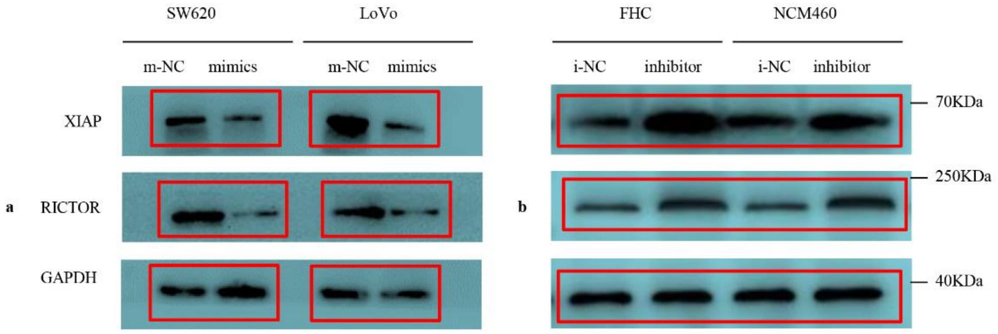

Supplementary Fig. 15 Original Western blotting images of the Supplementary Fig 4b and Supplementary Fig 7.

Supplementary Table 1. List of the oligonucleotides primers used for amplification

Experiment	Name	Position or orientation	Sequence (5'-3')
q-RT-PCR	CLCN5	F	CATTCTGACCGCCAGGGAAC
		R	CCCGCATTTGGTCATTCAGG
	HDAC2	F	AGACTGCAGTTGCCCTTGAT
		R	TGCGCAAATTTTCAAACAAA
	YY1	F	GAAGCCCTTTCAGTGCACGTT
		R	ACATAGGGCCTGTCTCCGGTAT
	GAPDH	F	AAATCCCATCACCATCTTCC
		R	TCACACCCATGACGAACA
	mature miR-500a-5p	RT	CTCAACTGGTGTCGTGGAGTCGGCAATT CAGTTGAGTCTCTCCC
		R	ACACTCCAGCTGGGTAATCCTTGCTACCTG G
	precursor miR-500a-5p	F	ATCCTTGCTACCTGGGTGAGA
		R	GCTCTCGCTCTCAGAATCCTT
	Primary miR-500a-5p	F	TGAGCCCTCTCACTGAACATT
		R	CTCTCACCCAGGTAGCAAGG
	snRNA U6	F	CTCGCTTCGGCAGCACATATA
		R	AACGCTTCACGAATTTGCGT
Luciferase Constructs (miR-500a-5p promoter)	R1	-5 ~ +16	R: 5' - GAAGATCT TGTTCTGCTCCCCCTCTCTA (Bgl II) -3'
	Site 1	-333 ~ -327	F: 5' - GGGGTACC TCA**<u>GATGAT</u>**TCTGGTGCATGT (KPN I) -3' *

	R2	-368 ~ -341	R: 5’ - GAAGATCT GACACCATTTAAGAGGTAGGCTTT (Bgl II) -3’
	Site 2	-628~ -622	F: 5’- GGGGTACC GAC**ACCATT**TAAGAGGTAGGCTTT (KPN I) -3’ *
	R3	-657 ~ -630	R: 5’ - GAAGATCT CAAGAAAATGAAATAAAGTATAGTACC (Bgl II) -3’
	Site 3	-747 ~ -741	F: 5’- GGGGTACC **ACCATC**CCCCTTAAAAATGCA (KPN I) -3’ *
	R1	-5 ~ +16	R: 5’ - GAAGATCT TGTTCTGCTCCCCCTCTCTA (Bgl II) -3’
	p-Luc-1kb	-1003 ~ -979	F: 5’- GGGGTACC TCTCTCTCTCTGTCACACACACAC (KPN I) -3’
Chip (miR-500a-5p promoter)	Chip 1	F: -371 ~ -391	TCACCCCAGACTCACCAAGT
		R: -197 ~ -217	GAGGAGTGTGAAAGGGAGGA
	Chip 2	F: -662 ~ -682	TAAAAATATTGACGGCTGTG
		R: -504 ~ -524	ACGAACCTTGGAACAAATG
	Chip 3	F: -816 ~ -838	GATGAATCTGTGTATCCCCTTG
		R: -658 ~ -676	TGGCACACAGCCGTCAATA
	Distant region	-4280 ~ -4260	GATTTGCAGCCGATTCATTT
		-4071 ~ -4051	TCCCCTTTTCCTGCCTTATT
siRNA	p300	Sense	5′- AACCCCUCCUCUUCAGCACCA-3′
	HDAC2		5′- AAGCATCAGGATTCTGTTA -3′
	YY1		5′- CGAGGAUCAGAUUCUCAUC -3′

* Bold and underline: YY1 binding sites

Supplementary table 2. Correlation between miR-500a-5p expression and the clinicopathological parameters of CRC

Characteristitis	Case	miR-500a expression (%)		$\chi 2$ value	P value
		Low	High		
Sex					
Male	50	26(52.0%)	24(48.0%)	0.100	0.752
Female	31	15(48.4%)	16(51.6%)		
Age(yr)					
＜60	41	19(46.3%)	22(53.7%)	0.607	0.436
≥60	40	22(55.0%)	18(45.0%)		
Tumor size					
＜3cm	10	3(30.0%)	7(70.0%)	1.113	0.291
≥3cm	71	38(53.5%)	33(46.5%)		
Differentiation					
Well	6	1(16.7%)	5(83.3%)	9.121	0.007*
Moderate	65	31(47.7%)	34(52.3%)		
Poor	10	9(90.0%)	1(10.0%)		
Lymph node metastasis					
Present	29	25(86.2%)	4(13.8%)	22.89	<0.001*
Absent	52	16(30.8%)	36(69.2%)		
TNM stage(AJCC)					
0-II	46	13(28.3%)	33(71.7%)	21.287	<0.001*
III-III	35	28(80.0%)	7(20.0%)		

Supplementary table 3. Correlation between HDAC2 expression and the clinicopathological parameters of CRC

Characteristitis	Case	HDAC2 expression (%)		$\chi 2$ value	P value
		Low	High		
Sex					
Male	50	20(40.0%)	30(60.0%)	4.601	0.032*
Female	31	20(64.5%)	11(35.5%)		
Age(yr)					
＜60	41	24(58.5%)	17(41.5%)	2.783	0.095
≥60	40	16(40.0%)	24(60.0%)		
Tumor size					
＜3cm	10	6(60.0%)	4(40.0%)	0.514	0.473
≥3cm	71	34(47.9%)	37(52.1%)		
Differentiantion					
Well	6	6(100%)	0(0.0%)	7.734	0.018*
Moderate	65	31(47.7%)	34(52.3%)		
Poor	10	3(30.0%)	7(70.0%)		
Lymph node metastasis					
Present	29	10 (34.5%)	19(65.5%)	4.012	0.045*
Absent	52	30(56.7%)	22(42.3%)		
TNM stage(AJCC)					
0-II	46	28(60.9%)	18(39.1%)	5.620	0.018*
III-VI	35	12(34.3%)	23(65.7%)		

Supplementary table 4. Correlation between YY1 expression and the clinicopathological parameters of CRC

Characteristitis	Case	YY1 expression (%)		$\chi 2$ value	P value
		Low	High		
Sex					
Male	50	24(48.0%)	26(52.0%)	0.1	0.752
Female	31	16(51.6%)	15(48.4%)		
Age(yr)					
＜60	41	19(46.3%)	22(53.7%)	0.307	0.579
≥60	40	21(52.5.0%)	19(47.5%)		
Tumor size					
＜3cm	10	8(80.0%)	2(20.0%)	2.995	0.084
≥3cm	71	32(45.1%)	39(54.9%)		
Differentiantion					
Well	6	6(100%)	0(0%)		0.004*
Moderate	65	32(49.2%)	33(52.8%)		
Poor	10	2(20.0%)	8(80.0%)		
Lymph node metastasis					
Present	29	7(24.1%)	22(75.9%)	11.517	0.001*
Absent	52	33(63.5%)	19(36.5%)		
TNM stage(AJCC)					
0-II	46	29(63.0%)	17(37.0%)	7.948	0.005*
III-III	35	11(31.4%)	24(68.6%)		

Supplementary table 5. HDAC2 interaction partners by LC-MS/MS analysis in LoVo cells

Gene Name	Accession	Score	Mass	Matches	emPAI	Protein description
MYH9	A0A024R1N1_HUMAN	5673	227646	255 (199)	12.69	Myosin, heavy polypeptide 9, non-muscle, isoform CRA_a
KRT1	K2C1_HUMAN	3052	66170	140 (109)	12.05	Keratin, type II cytoskeletal 1
HSPA8	HSP7C_HUMAN	1524	71082	60 (51)	7.36	Heat shock cognate 71 kDa protein
MCM5	B1AHB0_HUMAN	1339	83031	84 (60)	7.1	DNA helicase
MCM7	B2RBA6_HUMAN	1404	81856	69 (54)	6.72	DNA replication licensing factor MCM7
KRT2	K22E_HUMAN	1283	65678	65 (50)	5.71	Keratin, type II cytoskeletal 2 epidermal
KRT9	K1C9_HUMAN	1499	62255	81 (53)	4.75	Keratin, type I cytoskeletal 9
KRT10	K1C10_HUMAN	1047	59020	53 (39)	4.37	Keratin, type I cytoskeletal 10
PABPC1	A0A087WTT1_HUMAN	746	58727	45 (32)	4.13	Polyadenylate-binding protein
UBA52	M0R1V7_HUMAN	137	7128	9 (5)	4.06	Ubiquitin-60S ribosomal protein L40 (Fragment)
MTA2	A0A024R534_HUMAN	835	75717	53 (40)	4.01	Metastasis associated 1 family, member 2, isoform CRA_a
RT14	K1C14_HUMAN	602	51872	40 (28)	3.67	Keratin, type I cytoskeletal 14
KRT5	K2C5_HUMAN	552	62568	41 (32)	3.64	Keratin, type II cytoskeletal 5
EIF4G2	D3DQV9_HUMAN	965	102778	82 (59)	3.36	Eukaryotic translation initiation factor 4 gamma 2 (Fragment)
HSPA5	BIP_HUMAN	871	72402	41 (29)	2.62	Endoplasmic reticulum chaperone BiP
HSP90AB1	A0A024RD80_HUMAN	1034	83554	55 (39)	2.56	Heat shock protein 90kDa alpha (Cytosolic), class B member 1, isoform CRA_a
KRT17	K1C17_HUMAN	374	48361	37 (21)	2.5	Keratin, type I cytoskeletal 17
RAVER1	A0A087WZ13_HUMAN	701	78309	41 (32)	2.43	Ribonucleoprotein PTB-binding 1
DDX5	B5BUE6_HUMAN	591	69591	57 (27)	2.32	ATP-dependent RNA helicase DDX5 (Fragment)
HDAC2	HDAC2_HUMAN	364	55899	31 (21)	2.32	Histone deacetylase 2
SNW1	Q6I9S2_HUMAN	539	61468	42 (24)	2.31	SNW1 protein
KRT16	K1C16_HUMAN	486	51578	32 (20)	2.24	Keratin, type I cytoskeletal 16
ACTB	ACTB_HUMAN	345	42052	19 (16)	2.11	Actin, cytoplasmic 1
PPP1R13L	A0A024R0Q5_HUMAN	949	89378	48 (33)	2.05	Protein phosphatase 1, regulatory (Inhibitor) subunit 13 like, isoform CRA_a
TRIM29	A0A024R3J1_HUMAN	557	66478	40 (24)	2.03	Tripartite motif-containing 29, isoform CRA_a

PCK2	PCKGM_HUMAN	585	71452	41 (27)	1.94	Phosphoenolpyruvate carboxykinase [GTP], mitochondrial
DDX17	A0A1X7SBZ2_HUMAN	739	80888	39 (29)	1.92	Probable ATP-dependent RNA helicase DDX17
KRT6B	K2C6B_HUMAN	590	60315	42 (32)	1.89	Keratin, type II cytoskeletal 6B
DCD	DCD_HUMAN	93	11391	5 (4)	1.87	Dermcidin
PFKP	PFKAP_HUMAN	864	86454	42 (32)	1.83	ATP-dependent 6-phosphofructokinase, platelet type
TRAJ56	A0A075B6Z2_HUMAN	32	2220	27 (2)	1.81	T cell receptor alpha joining 56 (Fragment)
DDX3X	A0A0D9SF53_HUMAN	660	82110	40 (28)	1.77	ATP-dependent RNA helicase DDX3X
RBM14	A0A0S2Z4Z0_HUMAN	657	69620	36 (24)	1.76	RNA binding motif protein 14 isoform 1 (Fragment)
RPS6KA1	KS6A1_HUMAN	440	83070	36 (26)	1.74	Ribosomal protein S6 kinase alpha-1
MAGED2	A0A024R9Y7_HUMAN	686	65085	30 (22)	1.68	Melanoma antigen family D, 2, isoform CRA_a
ARHGEF7	A0A024RDY9_HUMAN	369	73664	32 (22)	1.61	Rho guanine nucleotide exchange factor (GEF) 7, isoform CRA_b
ASNS	ASNS_HUMAN	494	64899	38 (23)	1.55	Asparagine synthetase [glutamine-hydrolyzing]
HDAC1	HDAC1_HUMAN	313	55638	21 (17)	1.51	Histone deacetylase 1
FLJ11806	A0A024R6F2_HUMAN	447	49006	16 (14)	1.49	Nuclear protein UKp68, isoform CRA_a
CPS1	Q5R208_HUMAN	1136	165943	72 (48)	1.45	Carbamoylphosphate synthetase I
FUS	FUS_HUMAN	351	53622	15 (15)	1.45	RNA-binding protein FUS
CCT3	TCPG_HUMAN	351	61066	26 (16)	1.31	T-complex protein 1 subunit gamma
PROSER2	PRSR2_HUMAN	296	46116	19 (13)	1.29	Proline and serine-rich protein 2
RPS3	H0YEU2_HUMAN	123	18774	7 (5)	1.28	40S ribosomal protein S3 (Fragment)
CSTA	CYTA_HUMAN	78	11000	4 (3)	1.27	Cystatin-A
GIT1	A0A0C4DGN6_HUMAN	664	83821	32 (22)	1.24	ARF GTPase-activating protein GIT1
SEC13	C9J0S2_HUMAN	24	3138	1 (1)	1.24	Protein SEC13 homolog (Fragment)
UBC	F5H2Z3_HUMAN	110	15300	8 (4)	1.23	Polyubiquitin-C (Fragment)
ZC3H14	ZC3HE_HUMAN	579	83793	26 (20)	1.15	Zinc finger CCCH domain-containing protein 14
GATAD2A	A0A024R7K7_HUMAN	353	68363	26 (18)	1.12	GATA zinc finger domain containing 2A, isoform CRA_b
PABPC4	B1ANR0_HUMAN	248	68156	23 (16)	1.12	Polyadenylate-binding protein
AGPS	ADAS_HUMAN	415	73664	22 (17)	1.1	Alkyldihydroxyacetonephosphate synthase, peroxisomal
ALPL	A0A024RAG0_HUMAN	359	57611	23 (15)	1.06	Alkaline phosphatase
NCOR2	A0A024RBS3_HUMAN	1286	274461	97 (63)	1.05	Nuclear receptor co-repressor 2, isoform CRA_c

CLINT1	A0A0S2Z4Y4_HUMAN	442	68273	17 (15)	1.02	Clathrin interactor 1 isoform 1 (Fragment)
KRT14	K1C14_HUMAN	189	51872	14 (11)	0.97	Keratin, type I cytoskeletal 14
G3BP1	Q32P45_HUMAN	376	52155	34 (11)	0.96	GTPase activating protein (SH3 domain) binding protein 1
DDX1	A0A087X2G1_HUMAN	318	74898	25 (16)	0.9	ATP-dependent RNA helicase DDX1
NUSAP1	NUSAP_HUMAN	275	49593	18 (10)	0.9	Nucleolar and spindle-associated protein 1
PAPSS2	PAPS2_HUMAN	386	70027	17 (14)	0.9	Bifunctional 3'-phosphoadenosine 5'-phosphosulfate synthase 2
RPL13	A8K4C8_HUMAN	126	24304	9 (5)	0.9	60S ribosomal protein L13
ALB	ALBU_HUMAN	267	71317	20 (14)	0.88	Serum albumin
JUP	A0A024R1X8_HUMAN	360	82416	18 (16)	0.87	Junction plakoglobin, isoform CRA_a
CRTC3	CRTC3_HUMAN	529	67089	20 (14)	0.86	CREB-regulated transcription coactivator 3
SF1	A0A024R566_HUMAN	229	62135	18 (12)	0.86	Splicing factor 1, isoform CRA_a
SUGP1	SUGP1_HUMAN	458	72540	21 (14)	0.86	SURP and G-patch domain-containing protein 1
CCT6A	A0A024RDL1_HUMAN	183	58444	19 (11)	0.83	Chaperonin containing TCP1, subunit 6A (Zeta 1), isoform CRA_a
EEF2	EF2_HUMAN	357	96246	34 (18)	0.83	Elongation factor 2
GATAD2B	A0A0U1RRM1_HUMAN	360	63764	23 (15)	0.83	Transcriptional repressor p66-beta
KLC4	A0A024RCZ8_HUMAN	357	69054	23 (13)	0.83	Kinesin light chain 4, isoform CRA_a
MTA3	E7EQY4_HUMAN	176	59317	22 (11)	0.81	Metastasis-associated protein MTA3
TRIM25	D3DTY9_HUMAN	143	48358	15 (9)	0.81	Tripartite motif-containing 25, isoform CRA_a
PKP3	PKP3_HUMAN	314	87485	27 (17)	0.8	Plakophilin-3
KRT6A	A0A0S2Z428_HUMAN	273	60293	18 (11)	0.79	HCG2039812, isoform CRA_b (Fragment)
EI24	A0A087WV44_HUMAN	25	4801	3 (1)	0.77	Etoposide-induced protein 2.4 homolog
HSP90AA1	HS90A_HUMAN	566	85006	30 (17)	0.76	Heat shock protein HSP 90-alpha
SLC3A2	J3KPF3_HUMAN	392	68230	18 (12)	0.76	4F2 cell-surface antigen heavy chain
HIST1H1D	H13_HUMAN	98	22336	25 (4)	0.75	Histone H1.3
HNRPK	A0A024R228_HUMAN	190	51472	20 (10)	0.75	Heterogeneous nuclear ribonucleoprotein K, isoform CRA_d
PPM1H	S5ZYJ2_HUMAN	18	4893	1 (1)	0.75	SRGAP1/PPM1H fusion protein
RARS	SYRC_HUMAN	216	76129	20 (13)	0.73	Arginine--tRNA ligase, cytoplasmic
CNOT4	A0A024R781_HUMAN	284	71427	16 (12)	0.72	CCR4-NOT transcription complex, subunit 4, isoform CRA_b
ABCF2	A0A090N7X1_HUMAN	191	71815	22 (12)	0.71	ATP-binding cassette, sub-family F (GCN20), member 2

TFRC	TFR1_HUMAN	385	85274	22 (14)	0.7	Transferrin receptor protein 1
EEF1A1	EF1A1_HUMAN	130	50451	12 (8)	0.66	Elongation factor 1-alpha 1
G3BP2	A0A024RDB2_HUMAN	224	50843	10 (8)	0.65	Ras-GTPase activating protein SH3 domain-binding protein 2, isoform CRA_b
HADHA	ECHA_HUMAN	293	83688	20 (13)	0.65	Trifunctional enzyme subunit alpha, mitochondrial
RPS16	M0QX76_HUMAN	45	5611	1 (1)	0.64	40S ribosomal protein S16 (Fragment)
NELFA	A0A0C4DFX9_HUMAN	261	58691	16 (9)	0.63	Negative elongation factor A
TBL1XR1	A0A0D9SF63_HUMAN	174	52037	13 (8)	0.63	F-box-like/WD repeat-containing protein TBL1XR1
GFPT1	GFPT1_HUMAN	248	79555	17 (12)	0.62	Glutamine--fructose-6-phosphate aminotransferase [isomerizing] 1
DPYSL2	A0A1C7CYX9_HUMAN	392	74027	16 (12)	0.61	Dihydropyrimidinase-related protein 2
RCOR1	RCOR1_HUMAN	259	53409	10 (9)	0.61	REST corepressor 1
TCP1	TCPA_HUMAN	186	60819	15 (9)	0.61	T-complex protein 1 subunit alpha
BAG3	BAG3_HUMAN	365	61728	17 (9)	0.6	BAG family molecular chaperone regulator 3
CANX	CALX_HUMAN	294	67982	13 (10)	0.6	Calnexin
CDKN2AIP	CARF_HUMAN	370	61544	15 (9)	0.6	CDKN2A-interacting protein
EHD2	A0A024R0S6_HUMAN	121	61294	17 (10)	0.6	EH-domain containing 2, isoform CRA_a
HSPD1	A0A024R3X4_HUMAN	193	61187	13 (11)	0.6	Heat shock 60kDa protein 1 (Chaperonin), isoform CRA_a
RPN1	RPN1_HUMAN	286	68641	21 (10)	0.6	Dolichyl-diphosphooligosaccharide--protein glycosyltransferase subunit 1
FXR1	A0A0F7KYT8_HUMAN	285	76495	16 (11)	0.59	Fragile X mental retardation autosomal homolog variant p2K
KLC2	KLC2_HUMAN	211	69291	17 (10)	0.59	Kinesin light chain 2
BSG	BASI_HUMAN	142	42573	6 (6)	0.57	Basigin
CCT5	E9PCA1_HUMAN	132	57564	16 (8)	0.56	T-complex protein 1 subunit epsilon
RPS25	RS25_HUMAN	63	13791	3 (2)	0.56	40S ribosomal protein S25
ABCF3	A0A0S2Z5L1_HUMAN	247	80094	13 (11)	0.55	ATP-binding cassette sub-family F member 3 isoform 1 (Fragment)
CKAP4	A0A024RBH2_HUMAN	329	66097	14 (9)	0.55	Cytoskeleton-associated protein 4, isoform CRA_c
HSRP	A0A087WTP3_HUMAN	240	73438	15 (10)	0.55	Far upstream element-binding protein 2
FXR2	FXR2_HUMAN	215	74520	14 (10)	0.54	Fragile X mental retardation syndrome-related protein 2
PRRC2C	PRC2C_HUMAN	967	317346	73 (42)	0.54	Protein PRRC2C

RPRD2	RPRD2_HUMAN	661	156380	31 (21)	0.54	Regulation of nuclear pre-mRNA domain-containing protein 2
ABCE1	ABCE1_HUMAN	204	68240	18 (10)	0.53	ATP-binding cassette sub-family E member 1
AGFG1	A0A0S2Z3Y3_HUMAN	342	60213	14 (8)	0.53	ArfGAP with FG repeats 1 isoform 4 (Fragment)
ESS2	ESS2_HUMAN	201	52593	13 (7)	0.53	Splicing factor ESS-2 homolog
WNK1	WNK1_HUMAN	743	251552	48 (34)	0.53	Serine/threonine-protein kinase WNK1
GIT2	GIT2_HUMAN	206	85117	22 (11)	0.52	ARF GTPase-activating protein GIT2
IRF2BP2	I2BP2_HUMAN	414	61728	14 (8)	0.51	Interferon regulatory factor 2-binding protein 2
NONO	A0A0S2Z4Z9_HUMAN	112	54311	18 (8)	0.51	Non-POU domain containing octamer-binding isoform 1 (Fragment)
PFKL	PFKAL_HUMAN	342	85762	20 (11)	0.51	ATP-dependent 6-phosphofructokinase, liver type
RPL3	RL3_HUMAN	151	46365	8 (6)	0.51	60S ribosomal protein L3
ACADVL	ACADV_HUMAN	362	70745	16 (10)	0.5	Very long-chain specific acyl-CoA dehydrogenase, mitochondrial
MYH10	MYH10_HUMAN	591	229827	40 (29)	0.5	Myosin-10
MTA1	E7ESY4_HUMAN	174	80008	16 (10)	0.49	Metastasis-associated protein MTA1
RPL4	RL4_HUMAN	94	47953	12 (6)	0.49	60S ribosomal protein L4
TRIM25	TRI25_HUMAN	171	72581	14 (9)	0.49	E3 ubiquitin/ISG15 ligase TRIM25
CHD4	A0A0C4DGG9_HUMAN	349	221933	45 (27)	0.48	Chromodomain-helicase-DNA-binding protein 4
SDHA	A0A024QZ30_HUMAN	250	73672	15 (10)	0.48	Succinate dehydrogenase [ubiquinone] flavoprotein subunit, mitochondrial
RPL6	A0A024RBK3_HUMAN	83	32765	9 (4)	0.47	60S ribosomal protein L6
TMPO	LAP2A_HUMAN	274	76016	13 (10)	0.46	Lamina-associated polypeptide 2, isoform alpha
ZC3HAV1	ZCCHV_HUMAN	343	103135	15 (13)	0.45	Zinc finger CCCH-type antiviral protein 1
EIF3L	B0QY89_HUMAN	198	71085	13 (8)	0.44	Eukaryotic translation initiation factor 3 subunit L
QARS	SYQ_HUMAN	226	88655	17 (10)	0.44	Glutamine--tRNA ligase
ACSL3	A0A024R497_HUMAN	175	81338	13 (9)	0.43	Acyl-CoA synthetase long-chain family member 3, isoform CRA_a
TBL1X	A0A024RBV9_HUMAN	178	63255	12 (7)	0.43	Transducin (Beta)-like 1X-linked, isoform CRA_a
NSF	NSF_HUMAN	270	83055	19 (9)	0.42	Vesicle-fusing ATPase
hCG_1995254	A0A024R2J6_HUMAN	18	8595	1 (1)	0.41	HCG1995254, isoform CRA_a

HELLS	A0A0B4J1V9_HUMAN	171	103277	23 (11)	0.41	Helicase, lymphoid-specific, isoform CRA_b
RPS6KA3	KS6A3_HUMAN	165	84025	17 (9)	0.41	Ribosomal protein S6 kinase alpha-3
SPRR2F	SPR2F_HUMAN	32	8541	2 (1)	0.41	Small proline-rich protein 2F
CTPS1	PYRG1_HUMAN	218	67332	13 (7)	0.4	CTP synthase 1
KRT78	K2C78_HUMAN	100	57629	12 (6)	0.4	Keratin, type II cytoskeletal 78
LPP	LPP_HUMAN	135	67129	10 (7)	0.4	Lipoma-preferred partner
NCL	A0A024R4A0_HUMAN	214	76625	13 (8)	0.4	Nucleolin, isoform CRA_b
FLNA	FLNA_HUMAN	736	283301	56 (29)	0.39	Filamin-A
GRB10	GRB10_HUMAN	146	68158	11 (7)	0.39	Growth factor receptor-bound protein 10
MIER1	MIER1_HUMAN	178	58290	13 (6)	0.39	Mesoderm induction early response protein 1
SLC16A3	A0A024R8U1_HUMAN	109	50064	5 (5)	0.38	Solute carrier family 16 (Monocarboxylic acid transporters), member 3, isoform CRA_a
SORBS2	H7BXR3_HUMAN	184	70132	16 (7)	0.38	Sorbin and SH3 domain-containing protein 2 (Fragment)
SYNCRIP	HNRPQ_HUMAN	131	69788	13 (7)	0.38	Heterogeneous nuclear ribonucleoprotein Q
PAPSS1	PAPS1_HUMAN	152	71586	11 (7)	0.37	Bifunctional 3'-phosphoadenosine 5'-phosphosulfate synthase 1
RACGAP1	A0A024R136_HUMAN	231	71666	14 (8)	0.37	Rac GTPase activating protein 1, isoform CRA_a
SMAD4	A0A024R274_HUMAN	89	61199	9 (6)	0.37	Mothers against decapentaplegic homolog
COASY	COASY_HUMAN	191	62632	9 (6)	0.36	Bifunctional coenzyme A synthase
hCG_31253	A0A024R8A7_HUMAN	158	61944	10 (7)	0.36	HCG31253, isoform CRA_a
PRKAA1	AAPK1_HUMAN	175	64596	9 (6)	0.35	5'-AMP-activated protein kinase catalytic subunit alpha-1
RAVER2	RAVR2_HUMAN	98	74976	11 (7)	0.35	Ribonucleoprotein PTB-binding 2
TPX2	TPX2_HUMAN	192	86227	14 (8)	0.35	Targeting protein for Xklp2
NUMB	A0A024R681_HUMAN	162	66485	14 (6)	0.34	Numb homolog (Drosophila), isoform CRA_f
PRKDC	PRKDC_HUMAN	1033	473749	100 (43)	0.34	DNA-dependent protein kinase catalytic subunit
SPRR1B	SPR1B_HUMAN	32	10337	1 (1)	0.34	Cornifin-B
STIM1	STIM1_HUMAN	221	77660	12 (7)	0.34	Stromal interaction molecule 1
YY1	H0YJV7_HUMAN	139	21116	5 (2)	0.34	Transcriptional repressor protein YY1 (Fragment)
GTF2I	GTF2I_HUMAN	217	112859	18 (10)	0.33	General transcription factor II-I
MARS	SYMC_HUMAN	214	102249	14 (9)	0.33	Methionine--tRNA ligase, cytoplasmic

RPL18	J3QQ67_HUMAN	66	21940	5 (2)	0.33	60S ribosomal protein L18 (Fragment)
ATP6V1A	VATA_HUMAN	161	68660	9 (6)	0.32	V-type proton ATPase catalytic subunit A
PRDX1	A0A0A0MRQ5_HUMAN	44	10727	2 (1)	0.32	Peroxiredoxin-1
S100A8	S10A8_HUMAN	34	10885	2 (1)	0.32	Protein S100-A8
AARS	SYAC_HUMAN	290	107484	16 (9)	0.31	Alanine--tRNA ligase, cytoplasmic
CAT	CATA_HUMAN	156	59947	7 (5)	0.31	Catalase
PKM2	A0A024R5Z9_HUMAN	166	58538	9 (5)	0.31	Pyruvate kinase
PODXL	PODXL_HUMAN	112	59055	6 (5)	0.31	Podocalyxin
EL52	K9JA46_HUMAN	200	85006	13 (7)	0.3	Epididymis luminal secretory protein 52
LIMA1	LIMA1_HUMAN	161	85630	12 (7)	0.3	LIM domain and actin-binding protein 1
RPL15	A0A024R2Q4_HUMAN	58	24245	5 (2)	0.3	Ribosomal protein L15
S100A7	S10A7_HUMAN	44	11578	2 (1)	0.3	Protein S100-A7
SAMHD1	SAMH1_HUMAN	91	72896	14 (6)	0.3	Deoxynucleoside triphosphate triphosphohydrolase SAMHD1
TNKS1BP1	A0A024R542_HUMAN	456	182711	17 (15)	0.3	Tankyrase 1 binding protein 1, 182kDa, isoform CRA_a
ELP3	ELP3_HUMAN	137	62789	13 (5)	0.29	Elongator complex protein 3
LSM14A	LS14A_HUMAN	48	50727	9 (4)	0.29	Protein LSM14 homolog A
NUFIP2	NUFP2_HUMAN	297	76132	7 (6)	0.29	Nuclear fragile X mental retardation-interacting protein 2
ANKHD1-EIF4EBP3	H7C0V5_HUMAN	147	66378	7 (5)	0.27	ANKHD1-EIF4EBP3 readthrough (Fragment)
FUBP1	A0A1Z1G4M2_HUMAN	155	67733	8 (5)	0.27	FUBP1
LARP4B	LAR4B_HUMAN	159	80902	13 (6)	0.27	La-related protein 4B
ANKRD17	ANR17_HUMAN	471	275970	34 (20)	0.26	Ankyrin repeat domain-containing protein 17
KPNB1	IMB1_HUMAN	162	98420	11 (7)	0.26	Importin subunit beta-1
SLC7A5	LAT1_HUMAN	174	55659	7 (4)	0.26	Large neutral amino acids transporter small subunit 1
CAD	PYR1_HUMAN	334	245167	39 (17)	0.25	CAD protein
EIF2B4	EI2BD_HUMAN	84	58035	7 (4)	0.25	Translation initiation factor eIF-2B subunit delta
NSUN2	NSUN2_HUMAN	164	87214	15 (6)	0.25	tRNA (cytosine(34)-C(5))-methyltransferase
VPS33B	A0A0S2Z577_HUMAN	106	71225	14 (5)	0.25	Vacuolar protein sorting 33-like protein B isoform 1 (Fragment)
WRNIP1	A0A024RAG0_HUMAN	89	57611	5 (4)	0.25	Alkaline phosphatase
CUL4A	CUL4A_HUMAN	172	88138	13 (6)	0.24	Cullin-4A
HIST1H2BL	H2B1L_HUMAN	44	13944	3 (1)	0.24	Histone H2B type 1-L

HIST1H2BM	H2B1M_HUMAN	73	13981	3 (1)	0.24	Histone H2B type 1-M
SLAIN2	D6RIF6_HUMAN	41	44743	7 (3)	0.24	SLAIN motif-containing protein 2
MAP3K20	M3K20_HUMAN	282	91725	11 (7)	0.23	Mitogen-activated protein kinase kinase kinase 20
RPL14	A0PJ62_HUMAN	50	14716	2 (1)	0.23	RPL14 protein (Fragment)
TAF15	RBP56_HUMAN	186	62021	6 (4)	0.23	TATA-binding protein-associated factor 2N
YX	ZYX_HUMAN	60	62436	7 (4)	0.23	Zyxin
CD44	H0Y2P0_HUMAN	65	31332	4 (2)	0.22	CD44 antigen (Fragment)
EWSR1	B0QYK0_HUMAN	99	65174	5 (4)	0.22	RNA-binding protein EWS
IGF2BP1	D3DTW3_HUMAN	53	48111	8 (3)	0.22	Insulin-like growth factor 2 mRNA binding protein 1 deltaN CRDBP
SEPT9	SEPT9_HUMAN	121	65646	7 (5)	0.22	Septin-9
DTX3L	DTX3L_HUMAN	183	84585	13 (6)	0.21	E3 ubiquitin-protein ligase DTX3L
TCF25	H3BS87_HUMAN	30	15982	4 (1)	0.21	Transcription factor 25 (Fragment)
XRCC5	XRCC5_HUMAN	125	83222	12 (6)	0.21	X-ray repair cross-complementing protein 5
SMAD2	SMAD1_HUMAN	73	53025	3 (3)	0.2	Mothers against decapentaplegic homolog 1
TNPO1	A0A024RAM0_HUMAN	132	103771	13 (6)	0.2	Transportin 1, isoform CRA_a
TUBGCP3	GCP3_HUMAN	184	104304	11 (6)	0.2	Gamma-tubulin complex component 3
ABCB7	A0A2R8Y3N2_HUMAN	29	17550	3 (1)	0.19	ATP-binding cassette sub-family B member 7, mitochondrial (Fragment)
LMNA	LMNA_HUMAN	213	74380	15 (5)	0.19	Prelamin-A/C
NAP1L4	E9PP22_HUMAN	45	17453	2 (1)	0.19	Nucleosome assembly protein 1-like 4 (Fragment)
ATP1A1	AT1A1_HUMAN	201	114135	14 (6)	0.18	Sodium/potassium-transporting ATPase subunit alpha-1
DAK	A0A024R529_HUMAN	83	59282	6 (3)	0.18	Dihydroxyacetone kinase 2 homolog (Yeast), isoform CRA_a
DUSP28	DUS28_HUMAN	21	18654	2 (1)	0.18	Dual specificity phosphatase 28
EDC3	EDC3_HUMAN	70	56784	8 (3)	0.18	Enhancer of mRNA-decapping protein 3
AP2B1	A0A087X253_HUMAN	131	101953	12 (5)	0.17	AP complex subunit beta
CD55	H7BY55_HUMAN	105	59899	11 (4)	0.17	Complement decay-accelerating factor
CUL4B	CUL4B_HUMAN	81	104486	13 (5)	0.17	Cullin-4B
EIF2AK2	E2AK2_HUMAN	77	62512	3 (3)	0.17	Interferon-induced, double-stranded RNA-activated protein kinase
ATP5MF-PTCD1	G3V325_HUMAN	85	84684	10 (4)	0.16	ATP5MF-PTCD1 readthrough
COPB1	COPB_HUMAN	193	108214	14 (5)	0.16	Coatomer subunit beta
DDX21	DDX21_HUMAN	75	87804	13 (4)	0.16	Nucleolar RNA helicase 2

IGF2BP3	IF2B3_HUMAN	95	64008	12 (4)	0.16	Insulin-like growth factor 2 mRNA-binding protein 3
CPSF7	J3QT54_HUMAN	31	22606	2 (1)	0.15	Cleavage and polyadenylation-specificity factor subunit 7 (Fragment)
CSDE1	A0A024R0E2_HUMAN	67	89684	7 (4)	0.15	Cold shock domain containing E1, RNA-binding, isoform CRA_a
DHX15	DHX15_HUMAN	97	91673	10 (4)	0.15	Pre-mRNA-splicing factor ATP-dependent RNA helicase DHX15
MCM3	A0A0S2Z492_HUMAN	126	94633	11 (4)	0.15	DNA helicase
MIER2	A0A087WZA4_HUMAN	34	22628	1 (1)	0.15	Mesoderm induction early response protein 2 (Fragment)
MSN	MOES_HUMAN	47	67892	6 (3)	0.15	Moesin
HF21A	PF21A_HUMAN	165	75434	4 (3)	0.14	PHD finger protein 21A
HNRNPR	HNRPR_HUMAN	84	71184	5 (3)	0.14	Heterogeneous nuclear ribonucleoprotein R
ILF3	A0A024R7C7_HUMAN	76	95678	8 (4)	0.14	Interleukin enhancer binding factor 3, 90kDa, isoform CRA_d
PHF21A	PF21A_HUMAN	236	75434	6 (3)	0.14	PHD finger protein 21A
GNE	GLCNE_HUMAN	67	80193	10 (3)	0.13	Bifunctional UDP-N-acetylglucosamine 2-epimerase/N-acetylmannosamine kinase
GPBP1L1	GPBL1_HUMAN	61	52612	3 (2)	0.13	Vasculin-like protein 1
HLX	HLX_HUMAN	27	51043	6 (2)	0.13	H2.0-like homeobox protein
KRT80	K2C80_HUMAN	89	51007	5 (3)	0.13	Keratin, type II cytoskeletal 80
POLD3	A0A024R5M8_HUMAN	98	51653	5 (2)	0.13	Polymerase (DNA-directed), delta 3, accessory subunit, isoform CRA_a
HMMR	HMMR_HUMAN	147	84448	10 (3)	0.12	Hyaluronan mediated motility receptor
TAB1	TAB1_HUMAN	68	54895	4 (2)	0.12	TGF-beta-activated kinase 1 and MAP3K7-binding protein 1
TARS	SYTC_HUMAN	92	84294	12 (3)	0.12	Threonine--tRNA ligase, cytoplasmic
TBK1	TBK1_HUMAN	90	84216	12 (3)	0.12	Serine/threonine-protein kinase TBK1
VCL	A0A024QZN4_HUMAN	76	117220	11 (4)	0.12	Vinculin, isoform CRA_c
XP32	XP32_HUMAN	40	28557	1 (1)	0.12	Skin-specific protein 32
YWHAZ	D0PNI1_HUMAN	62	27899	1 (1)	0.12	Epididymis luminal protein 4
AHNAK	AHNK_HUMAN	421	629213	70 (21)	0.11	Neuroblast differentiation-associated protein AHNAK
DNAJC21	DJC21_HUMAN	162	62445	5 (2)	0.11	DnaJ homolog subfamily C member 21
DYNC1H1	DYHC1_HUMAN	276	534809	50 (18)	0.11	Cytoplasmic dynein 1 heavy chain 1
EHD1	A0A024R571_HUMAN	33	61945	8 (2)	0.11	EH domain-containing protein 1

ELAC2	A0A0S2Z5M8_HUMAN	41	93415	6 (3)	0.11	ElaC homolog 2 (E. coli), isoform CRA_b (Fragment)
HM13	A0A075B6F6_HUMAN	24	30314	1 (1)	0.11	Minor histocompatibility antigen H13 (Fragment)
ACAD9	ACAD9_HUMAN	40	69344	5 (2)	0.1	Acyl-CoA dehydrogenase family member 9, mitochondrial
CCDC174	A0A087WYA9_HUMAN	50	33679	4 (1)	0.1	Coiled-coil domain-containing protein 174
CDC23	CDC23_HUMAN	34	69588	5 (2)	0.1	Cell division cycle protein 23 homolog
CDYL	CDYL_HUMAN	56	66953	3 (2)	0.1	Chromodomain Y-like protein
CHD3	CHD3_HUMAN	164	227989	14 (7)	0.1	Chromodomain-helicase-DNA-binding protein 3
COPA	COPA_HUMAN	107	139797	10 (4)	0.1	Coatomer subunit alpha
C2CD2L	C2C2L_HUMAN	86	76590	5 (2)	0.09	Phospholipid transfer protein C2CD2L
CAPRIN1	CAPR1_HUMAN	96	78489	2 (2)	0.09	Caprin-1
CRTC2	CRTC2_HUMAN	96	73484	5 (2)	0.09	CREB-regulated transcription coactivator 2
DENND4C	A0A0A0MRX3_HUMAN	18	38395	2 (1)	0.09	DENN domain-containing protein 4C (Fragment)
LETM1	LETM1_HUMAN	82	83986	7 (2)	0.08	Mitochondrial proton/calcium exchanger protein
MCCC1	A0A0S2Z693_HUMAN	56	80935	6 (2)	0.08	Methylcrotonoyl-CoA carboxylase 1 isoform 1 (Fragment)
MISP3	A0A044PY82_HUMAN	36	39152	2 (1)	0.08	Uncharacterized protein MISP3
MRE11	F8W7U8_HUMAN	59	80814	7 (2)	0.08	Double-strand break repair protein MRE11
NCKIPSD	SPN90_HUMAN	53	79651	4 (2)	0.08	NCK-interacting protein with SH3 domain
ENO1	A0A024R4F1_HUMAN	30	47481	2 (1)	0.07	Enolase 1, (Alpha), isoform CRA_a
EXOSC9	A5PLM5_HUMAN	27	49515	3 (1)	0.07	Exosome component 9
HRNR	HORN_HUMAN	171	283140	12 (6)	0.07	Hornerin
LAMP1	A0A024RDY3_HUMAN	49	45367	1 (1)	0.07	Lysosomal-associated membrane protein 1, isoform CRA_a
CDADC1	CDAC1_HUMAN	22	59330	5 (1)	0.06	Cytidine and dCMP deaminase domain-containing protein 1
CDR2	CDR2_HUMAN	63	52165	3 (1)	0.06	Cerebellar degeneration-related protein 2
CNOT2	F8VV52_HUMAN	29	58754	4 (1)	0.06	CCR4-NOT transcription complex subunit 2 (Fragment)
DCTN4	A0A0S2Z5B8_HUMAN	32	51343	2 (1)	0.06	Dynactin 4 isoform 2 (Fragment)
EED	EED_HUMAN	41	50907	1 (1)	0.06	Polycomb protein EED

EXOC2	A0A024QZT2_HUMAN	65	105084	6 (2)	0.06	Exocyst complex component 2, isoform CRA_a
BMP2K	BMP2K_HUMAN	44	129947	5 (2)	0.05	BMP-2-inducible protein kinase
CDC73	CDC73_HUMAN	21	60653	7 (1)	0.05	Parafibromin
CDK5RAP1	A0A0S2Z5J9_HUMAN	60	66959	3 (1)	0.05	CDK5 regulatory subunit associated protein 1 isoform 2 (Fragment)
CHAF1B	CAF1B_HUMAN	34	61910	1 (1)	0.05	Chromatin assembly factor 1 subunit B
EIF3S9	A0A024R821_HUMAN	59	92823	4 (1)	0.04	Eukaryotic translation initiation factor 3 subunit B
ELOA	A0A024RAC6_HUMAN	46	87575	2 (1)	0.04	Elongin-A
ACTN4	A0A0S2Z3G9_HUMAN	62	105245	1 (1)	0.03	Actinin alpha 4 isoform 1 (Fragment)
AHNAK2	AHNK2_HUMAN	97	617383	21 (5)	0.03	Protein AHNAK2
ARHGEF2	D3DVA5_HUMAN	38	116954	4 (1)	0.03	Rho/rac guanine nucleotide exchange factor (GEF) 2, isoform CRA_a
ATP2A3	AT2A3_HUMAN	82	115444	6 (1)	0.03	Sarcoplasmic/endoplasmic reticulum calcium ATPase 3
HDAC7	HDAC7_HUMAN	90	103604	5 (1)	0.03	Histone deacetylase 7
HSPG2	A0A024RAB6_HUMAN	68	474443	11 (3)	0.02	Heparan sulfate proteoglycan 2 (Perlecan), isoform CRA_b
INF2	INF2_HUMAN	39	136851	5 (1)	0.02	Inverted formin-2
JMJD1C	JHD2C_HUMAN	90	286401	13 (2)	0.02	Probable JmjC domain-containing histone demethylation protein 2C
LARS	SYLC_HUMAN	26	135577	4 (1)	0.02	Leucine--tRNA ligase, cytoplasmic
LTBP1	LTBP1_HUMAN	49	195112	1 (1)	0.02	Latent-transforming growth factor beta-binding protein 1
MKI67	KI67_HUMAN	27	360698	18 (2)	0.02	Proliferation marker protein Ki-67
DMD	A0A075B6G3_HUMAN	54	428565	19 (1)	0.01	Dystrophin
DNAJC13	DJC13_HUMAN	32	256533	10 (1)	0.01	DnaJ homolog subfamily C member 13
EP300	EP300_HUMAN	31	266898	6 (1)	0.01	Histone acetyltransferase p300
GOLGA4	H0Y6I0_HUMAN	30	247368	13 (1)	0.01	Golgin subfamily A member 4 (Fragment)
HELZ2	HELZ2_HUMAN	26	298286	3 (1)	0.01	Helicase with zinc finger domain 2
LRBA	LRBA_HUMAN	35	321586	3 (1)	0.01	Lipopolysaccharide-responsive and beige-like anchor protein
MAP4	E7EVA0_HUMAN	44	246546	7 (1)	0.01	Microtubule-associated protein
TRIO	TRIO_HUMAN	77	349762	13 (2)	0.01	Triple functional domain protein
UTP20	UTP20_HUMAN	31	320805	7 (1)	0.01	Small subunit processome component 20 homolog
